# Mcm10 Self-Association Is Mediated by an N-Terminal Coiled-Coil Domain

**DOI:** 10.1371/journal.pone.0070518

**Published:** 2013-07-23

**Authors:** Wenyue Du, Ajeetha Josephrajan, Suraj Adhikary, Timothy Bowles, Anja-Katrin Bielinsky, Brandt F. Eichman

**Affiliations:** 1 Department of Biological Sciences, Vanderbilt University, Nashville, Tennessee, United States of America; 2 Department of Biochemistry, Molecular Biology and Biophysics, University of Minnesota, Minneapolis, Minnesota, United States of America; Saint Louis University, United States of America

## Abstract

Minichromosome maintenance protein 10 (Mcm10) is an essential eukaryotic DNA-binding replication factor thought to serve as a scaffold to coordinate enzymatic activities within the replisome. Mcm10 appears to function as an oligomer rather than in its monomeric form (or rather than as a monomer). However, various orthologs have been found to contain 1, 2, 3, 4, or 6 subunits and thus, this issue has remained controversial. Here, we show that self-association of *Xenopus laevis* Mcm10 is mediated by a conserved coiled-coil (CC) motif within the N-terminal domain (NTD). Crystallographic analysis of the CC at 2.4 Å resolution revealed a three-helix bundle, consistent with the formation of both dimeric and trimeric Mcm10 CCs in solution. Mutation of the side chains at the subunit interface disrupted *in vitro* dimerization of both the CC and the NTD as monitored by analytical ultracentrifugation. In addition, the same mutations also impeded self-interaction of the full-length protein *in vivo*, as measured by yeast-two hybrid assays. We conclude that Mcm10 likely forms dimers or trimers to promote its diverse functions during DNA replication.

## Introduction

DNA replication is carried out by multi-protein factories that in eukaryotes are assembled in stages to regulate the timing of DNA synthesis within the cell cycle [1], [2], [3]. Pre-replicative complexes (pre-RCs) are assembled at origins during G1 and are composed of origin recognition complex (ORC), Cdc6, Cdt1, and an inactive form of the minichromosome maintenance (Mcm) 2–7 helicase. The pre-RC is activated at the onset of S-phase by Dbf4-dependent kinase (DDK) and cyclin-dependent kinase (CDK) activities [4]. In yeast, CDK phosphorylates Sld2 and Sld3 and facilitates their binding to Dpb11 [5], [6], [7] and DDK phosphorylates Mcm2 and Mcm4 [8], [9] to promote the assembly of additional factors. Ultimately, pre-RC activation leads to the loading of Cdc45 and GINS (Go-Ichi-Nii-San), which form a functional helicase (CMG) complex with Mcm2–7 [10], [11], [12], [13], [14], [15]. Unwinding of the origin is signified by loading of replication protein A (RPA), followed by recruitment of DNA polymerase α (pol α)-primase, which initiates DNA synthesis at the heads of the leading strands and each Okazaki fragment.

Mcm10 is a non-enzymatic protein that aids assembly and activation of the replisome and coordinates helicase and polymerase activities during elongation [16], [17], [18]. Mcm10 interacts with single- (ss) and double-stranded (ds) DNA [19], [20], [21], is loaded onto chromatin in early S-phase, and is essential for helicase activation [22], [23], [24] and the subsequent recruitment of replisome proteins, including RPA and pol α [4], [16], [25]. *Saccharomyces cerevisiae* Mcm10 (scMcm10) is required to maintain pol α on chromatin independently of Cdc45 [16], and both Mcm10 and the sister chromatid cohesion protein And-1/Ctf4 have been implicated in loading pol α onto chromatin and physically coupling pol α and Mcm2–7 [15], [16], [18], [26], [27]. Mcm10 from various organisms has been shown to interact physically with key proteins involved in initiation and elongation, including ORC [28], [29], Mcm2–7 [28], [30], [31], [32], pol α [16], [17], [20], [33], [34], proliferating cell nuclear antigen (PCNA) [35], And-1 [18] and the RecQ-like helicase RecQ4 [18], [36]. Mcm10 binds to the Sld2-like sequence of the human RecQ4 helicase, suggesting that it may regulate the phosphorylation of RecQ4 to facilitate initiation [36]. Furthermore, loss of Mcm10 from human cells causes chromosome breakage and genomic instability [3], [17].

Mcm10 contains at least three functional domains [20]. An N-terminal coiled-coil (CC) domain (NTD) has been implicated in Mcm10 self-association [20] and the interaction with Mec3, a subunit of the 9-1-1 clamp (Alver and Bielinsky, unpublished results). In addition, the protein has a highly conserved internal (ID) and vertebrate-specific C-terminal domain (CTD) that bind DNA and the catalytic (p180) subunit of pol α [20], [34], [37]. The yeast orthologs have also been shown to interact with DNA and pol α despite the apparent lack of the CTD [16], [19], [21], [38]. Thus, the ID is likely to mediate these interactions in *S. cerevisiae*. Moreover, recent evidence suggests that acetylation of the ID and CTD in human Mcm10 differentially controls their respective DNA binding and protein-protein interactions [39]. However, the details of this potential mechanism are still unclear.

The oligomeric state of Mcm10 has remained controversial, with reports ranging in size from 1–12 subunits [37]. scMcm10 was shown by size-exclusion chromatography to form large, 800-kDa homocomplexes consisting of ∼12 molecules [40]. Self-association in that case was presumably dependent on the integrity of the zinc-finger (ZnF) motif within the ID, although purified ID from *Xenopus laevis* Mcm10 (xMcm10) was found to be monomeric [20]. Electron microscopy (EM) and single-particle analysis of the human protein showed a hexameric ring-shaped structure [41]. In contrast, asymmetric monomeric and dimeric forms of *S. pombe* Mcm10 (spMcm10) were reported [42], [43]. Similarly, xMcm10 exhibited mass-dependent association into low molecular weight complexes that were presumed to represent Mcm10 dimers solely on the basis of dimerization of the isolated NTD [20]. Consistent with NTD-mediated self-assembly, scMcm10 showed a strong yeast two-hybrid interaction that was ablated when one binding partner carried a truncation of the first 100 amino acids. Moreover, these truncation mutants exhibited a striking sensitivity to the replication inhibitor hydroxyurea that was revealed in the absence of the 9-1-1 checkpoint clamp (Alver and Bielinsky, unpublished results). These observations agree with a report that demonstrates that scMcm10 is monomeric when bound to dsDNA, but capable of forming multi-subunit complexes on ssDNA [21].

Here, we studied the role of the NTD on xMcm10 self-assembly using structural, biophysical, and *in vivo* binding assays. We show that the CC region is necessary and sufficient to explain Mcm10-Mcm10 interaction, and is capable of forming both dimers and trimers in solution. The trimeric form of the CC was stabilized in a crystal structure, which revealed the residues at the subunit interface. Specific mutations at this interface disrupted dimerization of the isolated CC, the NTD, and eliminated self-association of the full-length protein by yeast-two hybrid interaction.

## Materials and Methods

### Protein Purification

Full-length xMcm10 was purified from baculovirus infected insect cells using the Bac-to-Bac expression system (Invitrogen). The gene was subcloned into pFastBac1 vector with a His_6_ tag added to the C-terminus by PCR. Protein was expressed in Hi-5 insect cells for 48 hr. Cells were resuspended in lysis buffer (50 mM Tris buffer (pH 7.5), 500 mM NaCl, 10% glycerol) and hand homogenized. Protein was purified by nickel-nitrilotriacetic (NTA) acid affinity chromatography. Pooled Ni-NTA fractions were buffer exchanged into 50 mM Tris buffer (pH 7.5), 150 mM NaCl, and 10% glycerol and purified using Source Q (GE Healthcare) cation exchange, followed by gel filtration on a Superose6 (GE Healthcare) column equilibrated in 25 mM Tris buffer (pH 7.5), 150 mM NaCl, 5% glycerol, and 2mM β-mercaptoethanol (BME). Mcm10ΔN (aa 230–860) and Mcm10-NTD (aa 1–145) were expressed and purified as previously described [20], [34].

Gene sequences encoding xMcm10 amino acids 95–124 and 95–132 were cloned into a pMALX(E) vector using NotI and BamHI restriction sites to generate CC fragments fused to the C-terminal end of a mutant form of maltose binding protein (MBP) with a short, uncleavable peptide linker as previously described [44]. The recombinant proteins were overexpressed in *E.coli* C41 cells for 3 hrs at 37°C in LB medium supplemented with 100 µg/mL ampicillin with addition of 0.5 mM IPTG at mid-log phase. Cells were resuspended in lysis buffer and lysed under pressure (25,000 p.s.i.) using an EmulsiFlex-C3 homogenizer (Avestin, Inc.). Lysate was centrifuged at 35,000×g for 20 min. The supernatant was incubated with amylose resin (New England Biolabs) overnight at 4°C and washed with 15 column volumes of lysis buffer. Fusion proteins were eluted with 40 mM maltose in lysis buffer, concentrated, and further purified by size exclusion chromatography on a Superdex S200 column (GE Healthcare) equilibrated in 25 mM Tris buffer (pH 7.5), 150 mM NaCl, 5% glycerol, 4 mM BME, and 40 mM maltose. Purified MBP-CC proteins were flash frozen and stored at -80°C in 25 mM Tris buffer (pH 7.5), 150 mM NaCl, 0.2 mM tris(2-carboxyethyl)phosphine hydrochloride (TCEP), and 5 mM maltose.

The 2A (L104A/L108A), 2D (L104D/L108D) and 4A (L104A/L108A/M115A/L118A) mutations were incorporated into full-length, NTD, and MBP-CC constructs using the QuikChange Mutagenesis Kit (Qiagen). Mutant proteins were expressed and purified the same as their corresponding wild-type proteins.

### X-ray Crystallography

Purified MBP-CC proteins were concentrated to 50 mg/mL using a 10-kDa MWCO Amicon spin concentrator and buffer exchanged into 25 mM Tris buffer (pH 7.5), 150 mM NaCl, 0.2 mM TCEP, and 5 mM maltose for crystallization. Crystals were grown by sitting drop vapor diffusion at 16°C by adding 2 µl protein to 2 µl reservoir solutions containing 0.1 M sodium acetate (pH 4.8), 0.1 M NaCl, 0.1 M CaCl_2_, 15% PEG 2K, and 5% (w/v) N-dodecyl-beta-D-maltoside (MBP-CC^95–124^), or 0.05 M sodium acetate (pH 4.8), 0.2 M NH_4_H_2_PO_4_, and 12% PEG 3350 (MBP-CC^95–132^). Crystals were flash frozen in 0.1 M sodium acetate (pH 4.8), 0.1 M NaCl, 0.1 M CaCl_2_, 22% PEG 2K, and 15% glycerol (MBP-CC^95–124^) or 0.05 M sodium acetate (pH 4.8), 0.2 M NH_4_H_2_PO_4_, 23% PEG 3350, and 15% glycerol (MBP-CC^95–132^) prior to data collection. X-ray diffraction data were collected at the Advanced Photon Source LS-CAT/sector 21 and processed using HKL2000 [45].

The structures of MBP-CC^95–124^ (2.4 Å) and MBP-CC^95–132^ (3.1 Å) were determined by molecular replacement using MBPX(E) from PDB ID 3H4Z as a search model [44], [46]. Phases generated from three copies of MBP in the asymmetric unit revealed clear electron density for the Mcm10 coiled-coil in both cases. The models were built in COOT [47] and refined against a maximum likelihood target in PHENIX [48]. Although one additional turn of the α-helix was visible in the MBP-CC^95–132^ structure, the side chains could not be unambiguously identified, and thus the lower resolution structure was not pursued further. Anisotropic motion was modeled using translation/libration/screw-rotation (TLS) refinement, with each protomer defined as a TLS group. Individual anisotropic B-factors derived from the refined TLS parameters were held fixed during subsequent rounds of refinement. Adjustments to the model and addition of solvent was carried out iteratively through inspection of 2F_o_-F_c_, F_o_-F_c_ and composite omit electron density maps. The final MBP-CC^95–124^ model, consisting of MBPX(E) residues 1–367, the five-residue linker (AAAMG), and xMcm10 residues 95–122, was validated using PROCHECK [49]. 97.5% and 2.2% of residues reside in the favored and allowed regions of the Ramachandran plot, respectively. The remaining 0.3% in disallowed regions reside in the MBP-CC linker, MBP loops, or the extreme MBP amino terminus. The final model was deposited in the Protein Data Bank under accession number 4JBZ.

### Ultracentrifugation and Light Scattering

Sedimentation velocity experiments were performed using a Beckman ProteomeLab XL-I ultracentrifuge operating at 42,000 rpm and 4°C (Mcm10, Mcm10ΔN) or 20°C (MBP, MBP-CC, and NTD). Full-length Mcm10 was concentrated to 1.6 mg/ml in PBS buffer (pH 7.4), 150 mM NaCl, and 0.3 mM TCEP, and Mcm10ΔN was analyzed at 1.0 mg/ml in 25 mM Tris buffer (pH 7.5), 150 mM NaCl, 2 mM MgCl_2_, 5% glycerol, and 4 mM BME. MBP, MBP-CC, and NTD constructs were analyzed at 0.6 mg/ml in PBS buffer (pH 7.4), 150 mM NaCl, and 0.3 mM TCEP or 25 mM sodium acetate buffer (pH 4.7), 150 mM NaCl, and 0.3 mM TCEP. Buffer viscosity, buffer density and partial specific volume were calculated using SEDNTERP [50]. Data was processed using c(s) analysis in SEDFIT [51], [52].

Molecular mass analysis of full-length Mcm10 and Mcm10ΔN by size exclusion chromatography and multi-angle light scattering (SEC-MALS) was carried out using a Superose6 10/300 GL column (GE Healthcare) operating at 0.4 ml/min in 25 mM Tris buffer (pH 7.5), 150 mM NaCl, 2% glycerol, and 2 mM BME. Absorbance, refractive index, and light scattering of the eluants were measured using a DAWN HELEOS II detector (Wyatt Technology) and data analyzed by ASTRA software.

### Yeast Two-hybrid Assay and Immunoblotting

The Matchmaker 3 (Clontech) system was utilized to measure protein-protein interaction by yeast two-hybrid assay. Genes encoding the bait proteins were cloned into pGBKT7 to express Myc-tagged fusions with the Gal4-binding domain. Genes encoding the prey proteins were cloned into pGADT7 to express hemagglutinin (HA)-tagged fusions with the Gal4- activation domain. Plasmids were sequence verified and transformed into the reporter strain, AH109 (Clontech), in which the expression of *ADE2* and *HIS3* was under the control of a *GAL1,10* promoter. Transformants were selected on medium lacking leucine and tryptophan, and protein interaction was scored on quadruple drop-out plates lacking adenine, leucine, histidine and tryptophan. To verify protein expression, total protein extracts were obtained from yeast cultures by trichloroacetic acid (TCA) preparation as described and separated by SDS-PAGE and subsequently transferred onto nitrocellulose membrane [53]. HA-tagged xMcm10 was visualized using a horseradish peroxidase (HRP)-conjugated anti-HA antibody (Roche, 3F10). Myc-tagged xMcm10 was detected using an anti-Myc antibody (Thermo Scientific, 9E11).

## Results

### Mcm10 Self-associates through its N-terminal Domain

**Figure 1:**
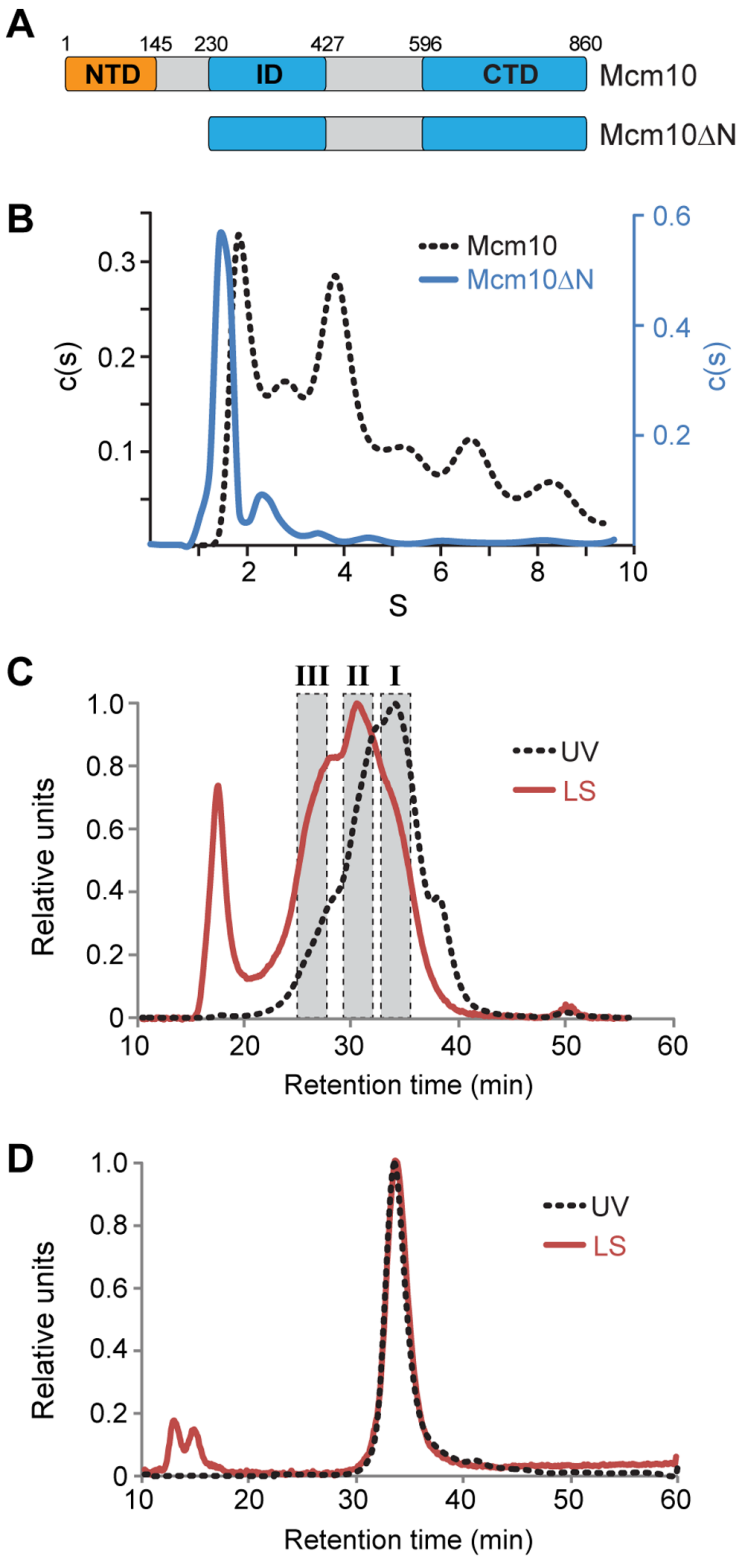
The NTD is necessary for Mcm10 self-association. (A) Schematic of *Xenopus laevis* Mcm10 constructs used to study self-association. Theoretical molecular masses are 95.4 kDa (Mcm10) and 70.4 kDa (Mcm10ΔN). (B) Sedimentation velocity data for full-length Mcm10 (black dotted line) and Mcm10ΔN (blue) as described in Materials and Methods. The molecular mass of the Mcm10ΔN major peak was calculated to be 68.8 kDa. Molecular masses could not be accurately determined from the full-length data. (C,D) SEC-MALS analysis of Mcm10 (C) and Mcm10ΔN (D). The UV trace is shown as a black dotted line and the light scattering trace is red. (C) Estimated molecular masses were calculated from the three shaded regions to be 90.4 kDa (I), 189.3 kDa (II), and 322.7 kDa (III). The peak at 18 min corresponds to the void volume. (D) The molecular mass of Mcm10ΔN was calculated to be 75.1±0.8 kDa.

Full-length xMcm10 self-associates into low molecular mass complexes, which we previously hypothesized to form as a result of NTD dimerization [20]. In order to investigate the contribution of the NTD on self-association behavior, we purified a deletion mutant lacking the first 230 residues of xMcm10 (Mcm10ΔN) and analyzed molecular masses of full-length and Mcm10ΔN proteins by sedimentation velocity analytical ultracentrifugation and size exclusion chromatography coupled to multi-angle light scattering (SEC-MALS). The full-length protein showed a broad distribution of low and high sedimenting species indicative of multiple oligomeric states (Figure 1B). The complex nature of the sedimentation profile precluded assignment of precise molecular mass to each peak. Similarly, SEC-MALS analysis of the full-length protein showed a broad elution profile with at least three overlapping but distinct peaks and the majority of the protein existing as the lower molecular weight form (Figure 1C). As with the sedimentation data, the overlapping nature of the peaks only allowed for an estimation – not a definitive assessment – of the respective molecular masses. From the light scattering data, the three major species were approximately 90.4 kDa (I), 189.3 kDa (II), and 322.7 kDa (III) in size, corresponding to 1, 2, and 3.4 Mcm10 subunits, respectively (the calculated mass from amino acid composition is 95.4 kDa). Although not strictly conclusive, these data are consistent with reports of dimeric and trimeric forms of yeast Mcm10 [21], [42].

In contrast, Mcm10ΔN formed a single species corresponding unequivocally to a monomeric protein in both experiments. The molecular mass of the major (1.5S) peak from sedimentation velocity (Figure 1B) was calculated to be 68.8 kDa, compared to 70.4 kDa calculated from the amino acid composition. The minor species observed at 2.3S did not increase with the protein concentration (Table S1) and was thus judged to be a contaminant. In addition, Mcm10ΔN eluted as a single, monodispersed species from a size exclusion column with a molecular mass of 75.1±0.8 kDa determined by MALS (Figure 1D). Therefore, deletion of the NTD eliminated self-association of the full-length protein.

### The Structure of the Mcm10 Coiled-coil Region

The NTD of the vertebrate and *Saccharomyces cerevisiae* Mcm10 orthologs contain a putative CC that we previously hypothesized accounts for dimerization of the NTD [20]. We tested the ability of this region to dimerize by fusing the peptide corresponding to xMcm10 residues 95–132 to maltose binding protein (MBP) and analyzing by sedimentation velocity ultracentrifugation. MBP alone sedimented as a monomer, in agreement with a previous determination [54]. In contrast, two species consistent with monomeric and dimeric forms of MBP-CC were present (Figure S1A,B). The dimeric form of MBP-CC persisted on SDS-PAGE gels even in the presence of high concentrations of reducing agents (Figure S1C), a characteristic of coiled-coils observed in other proteins [55].

**Figure 2:**
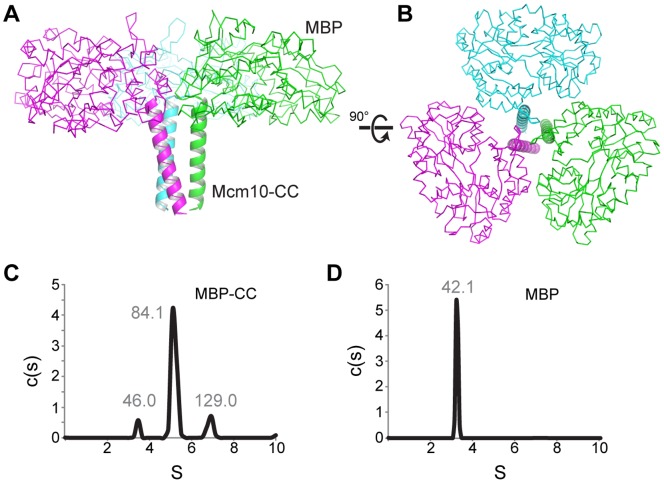
Trimerization of MBP-CC. (A,B) Crystal structure of the MBP-CC asymmetric unit, with each protomer colored differently. Maltose-binding protein is shown as a C_α_-trace, and the xMcm10 coiled-coil is depicted as a cartoon ribbon. (C,D) Sedimentation velocity profiles of MBP-CC^95–132^ (C) and free MBP (D) at pH 4.7. Molecular masses (kDa) calculated from the sedimentation data are shown above each peak. The molecular mass of a single polypeptide calculated from the amino acid composition are 45.1 kDa (MBP-CC) and 40.4 kDa (MBP).

To verify this region of the protein as a *bona fide* CC, we determined the crystal structure of the MBP-CC fusion protein to a resolution of 2.4 Å (Figure 2). The highest quality diffraction data were obtained from a construct spanning xMcm10 residues 95–124 crystallized under low pH conditions (Table S2). The final model was refined to crystallographic residuals of 16.4% (R_work_) and 20.5% (R_free_). Surprisingly, the asymmetric unit consisted of a trimeric assembly with the Mcm10 residues at the center forming a parallel three-helix CC wrapped in a left-handed superhelix (Figure 2A,B). Trimer formation is not a crystallographic artifact, since we observed trimeric and dimeric MBP-CCs in solution under the same (low pH) conditions used for crystallization (Figure 2C). Similarly, we verified that MBP did not influence trimerization since MBP alone is monomeric in solution at the low pH condition (Figure 2D). Thus, the Mcm10 CC has the propensity to form both dimeric and trimeric helical bundles, consistent with our SEC-MALS analysis of the full-length protein (Figure 1C).

**Figure 3:**
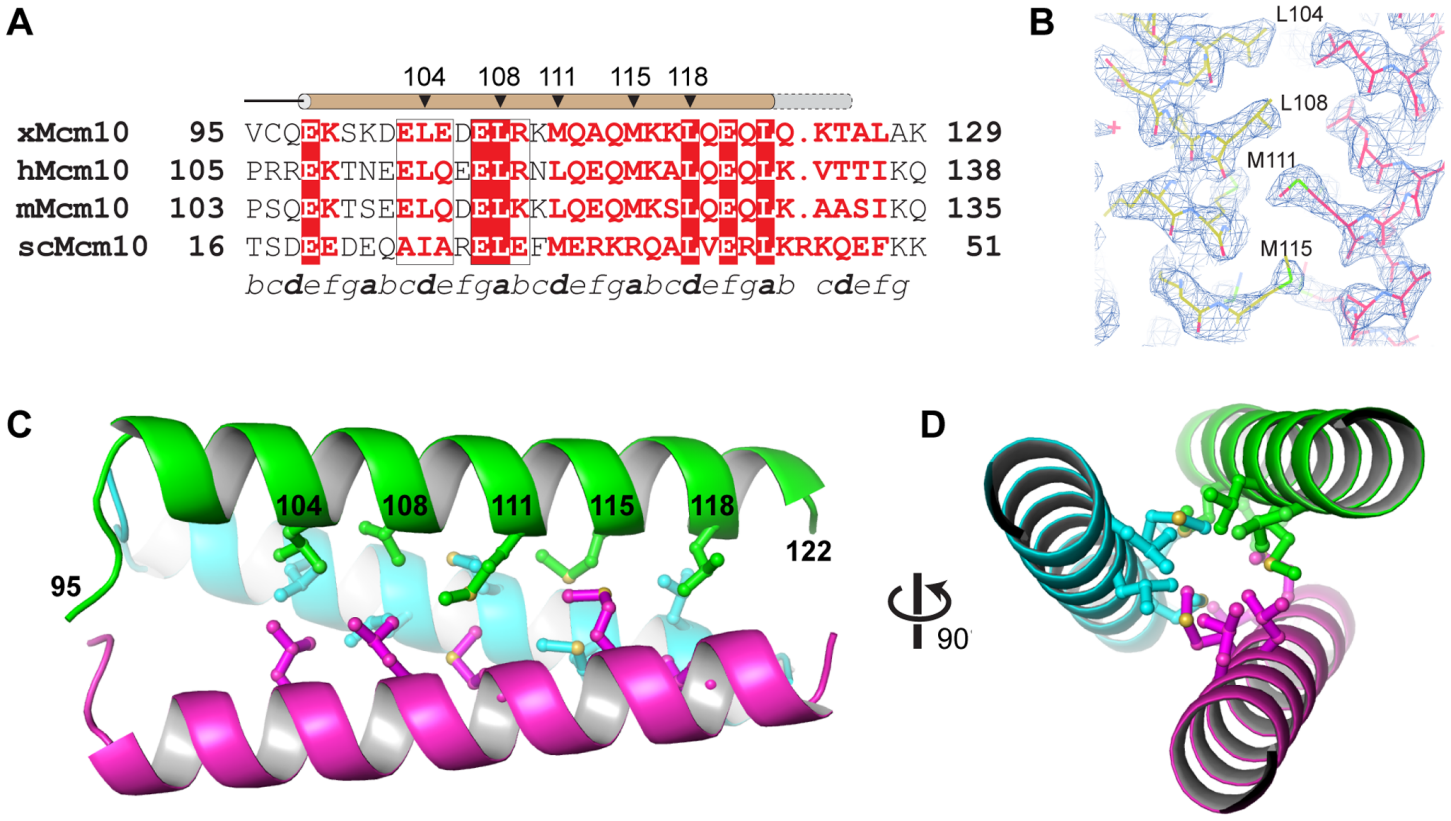
Crystal structure of the Mcm10 coiled-coil. (A) Sequence alignment of the coiled-coil region from *Xenopus laevis* (x), *Homo sapiens* (h), *Mus musculus* (m), and *Saccharomyces cerevisiae* (sc) Mcm10. Red boxes indicate identical amino acids. The predicted heptad repeat pattern is labeled by letters *a-g* at the bottom. The position of the helix is shown schematically at the top (brown, MBP-CC^95–124^; grey, MBP-CC^95–132^). (B) Composite 2F_o_−F_c_ omit electron density map (contoured at 1σ) with carbon atoms colored according to protomer. (C) Crystal structure of xMcm10-CC^95–124^. Residues at the interface are shown in ball and stick, and the amino acid numbers labeled in black. (D) View down the helical axis, rotated 90° from the view in *B*.

CCs in other proteins have been shown to exist in multiple oligomeric states, a property largely dependent on the characteristics of the *a* and *d* hydrophobic side chains of the heptad repeat that form the helical interface [56]. For example, two-, three-, and four-stranded CCs in the GCN4 leucine zipper were engineered by mutating the *a* and *d* positions [57]. The Mcm10 CC helical region spans Glu98 to Leu122, two invariant residues in the human, frog, mouse, and budding yeast orthologs (Figure 3A), although we did observe the helices to extend to at least Thr125 in lower resolution structures obtained from a longer 95–132 construct (data not shown). Most importantly, the high resolution of the structure enabled us to identify the residues of the CC interface as Leu104, Leu108, Met111, Met115, and Leu118 (Figure 3B). This interface is entirely hydrophobic, with the side chains of each residue interacting with its equivalents on the other two helices through van der Waals packing around a three-fold rotation axis (Figure 3C,D).

**Figure 4:**
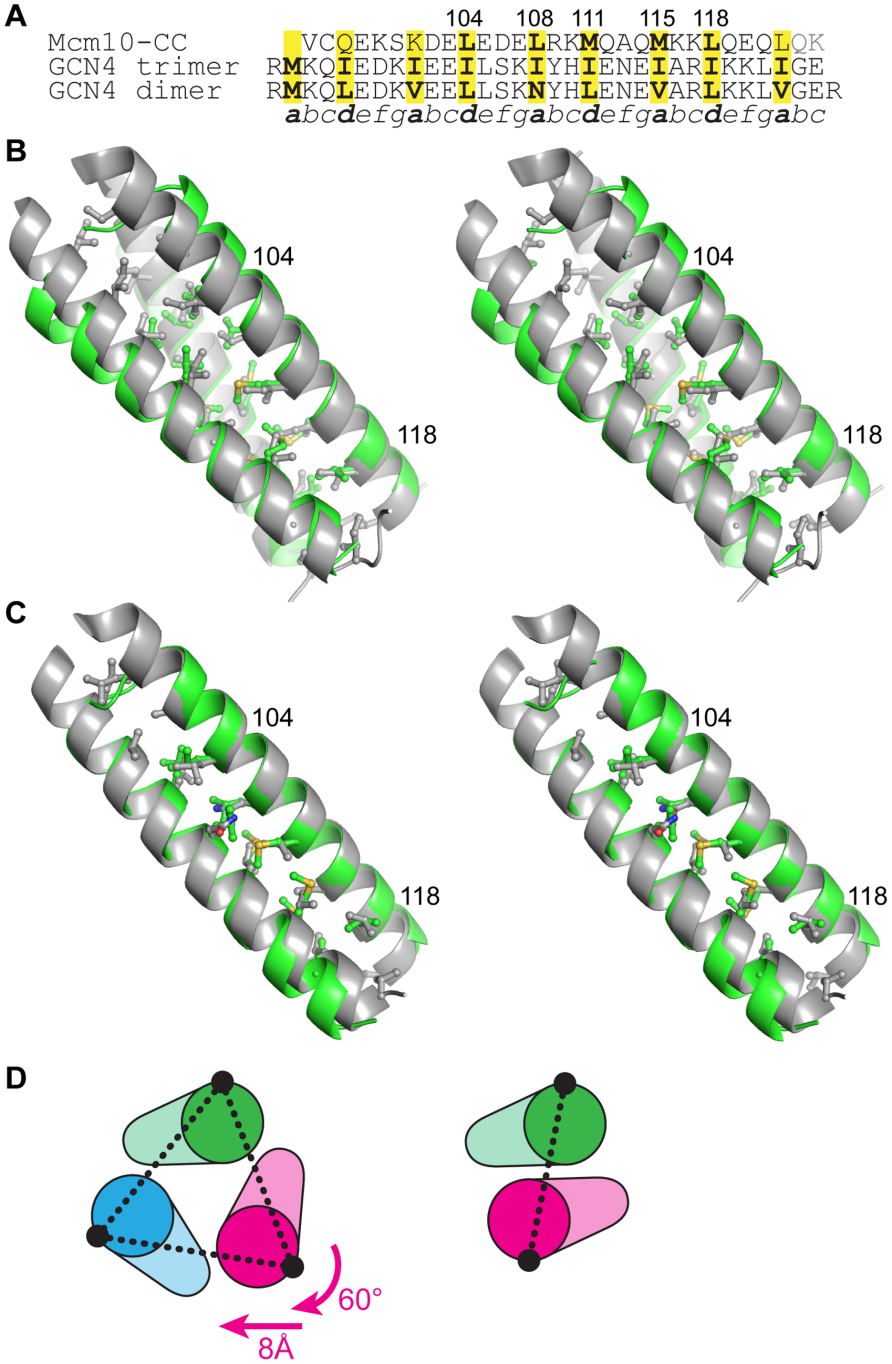
A model of the dimeric Mcm10 CC. (A) Structure-based sequence alignment between Mcm10 CC and the trimeric and dimeric forms of GCN4 CC. (B) Stereoview of the Mcm10 CC trimer (green) superimposed onto the isoleucine GCN4 trimer (grey, PDB ID 1 GCM). Side chains at the CC interface are shown, with the exception of the N-terminal methionine in GCN4. Two Mcm10 residues are labeled for orientation. The view is rotated 45° clockwise with respect to Figure 3C. (C) Stereoview of the Mcm10 CC dimer model (green) superimposed on the CGN4 CC (grey, PDB ID 2ZTA). (D) The relationship between trimer (left) and dimer (right) forms of the CC. Schematics are oriented with respect to the green helix. The conformation of the dimer can be constructed from the trimer by a 60° rotation and 8 Å translation of the magenta helix. The interhelical distances (dashed line) are 15.5 Å (trimer) and 10 Å (dimer).

We expected the same residues to form the interface in a dimeric form of the CC based on other structures with both dimeric and trimeric propensities [57], [58], [59]. The conformation of the Mcm10 CC trimer is virtually identical to the isoleucine zipper variant of the GCN4 CC [58], with only a modest divergence at the N-terminal end (Figure 4B), which likely results from non-hydrophobic heptad repeat *a* and *d* residues (Gln97, Lys101) and/or the MBP tag (Figure 4A). We therefore constructed a model of the dimeric Mcm10 CC using the GCN4 leucine zipper dimer as a template (Figure 4C) [60]. As shown in Figure 4D, the dimer and trimer are related by a simple 60° rotation and 8 Å translation of one helix. The *a* and *d* positions are conserved between the two models, and the conformations of only two side chains (Leu104 and Leu108) needed to be adjusted to avoid steric collision across the dimer interface. Thus, only modest adjustments are required to interconvert between the CC dimer and trimer.

### Mutations in the Coiled-coil Motif Disrupt Mcm10 Oligomerization

**Figure 5:**
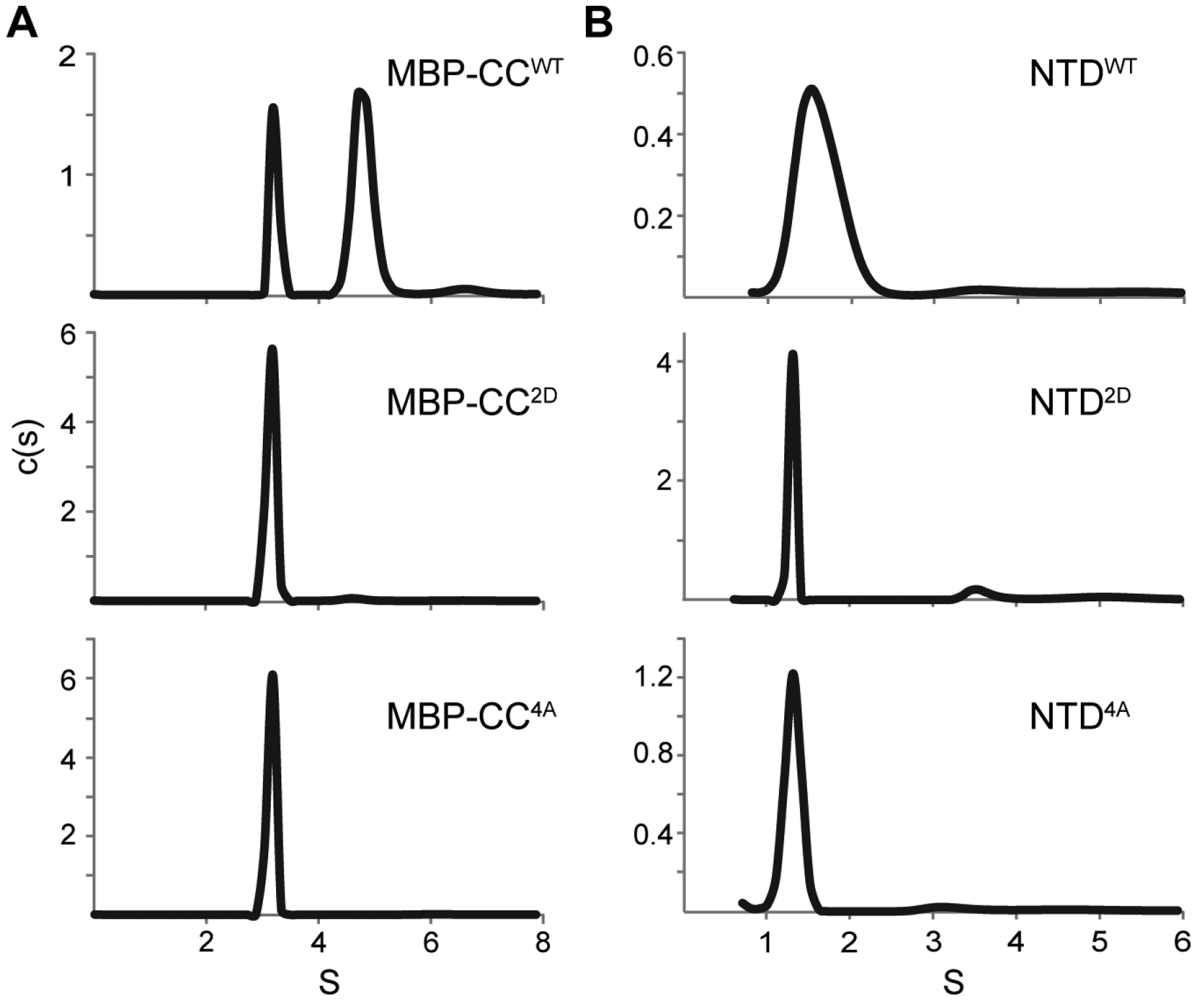
Coiled-coil mutations disrupt CC and NTD dimerization. Sedimentation velocity data for MBP-CC^95–124^ (A) and NTD (B) constructs as wild-type (WT) or containing L104D/L108D (2D) or L104A/L108A/M115A/L118A (4A) mutations. Molecular masses corresponding to each peak are reported in Table S1 in the Supporting Information.

To validate the crystal structure as representative of a functional CC, we designed mutations aimed at disrupting self-interaction. We introduced electrostatic repulsion at the interface by substituting Leu104 and Leu108 with aspartate to create a L104D/L108D double (2D) mutant. In addition, we eliminated side chains at positions 104, 108, 115, and 118 by alanine substitution to create a L104A/L108A/M115A/M118A quadruple (4A) mutant. Mutations were introduced into the MBP-CC^95–124^ and NTD protein constructs and tested for dimerization using sedimentation velocity (Figure 5). Both 2D and 4A mutants disrupted dimerization of the wild-type CC and NTD (Figure 5A,B). Interestingly, replacing only Leu104 and Leu108 with alanine (2A mutant) was not enough to disrupt dimerization (Figure S2A), suggesting that the remainder of the interface is sufficient to hold the CC together.

**Figure 6:**
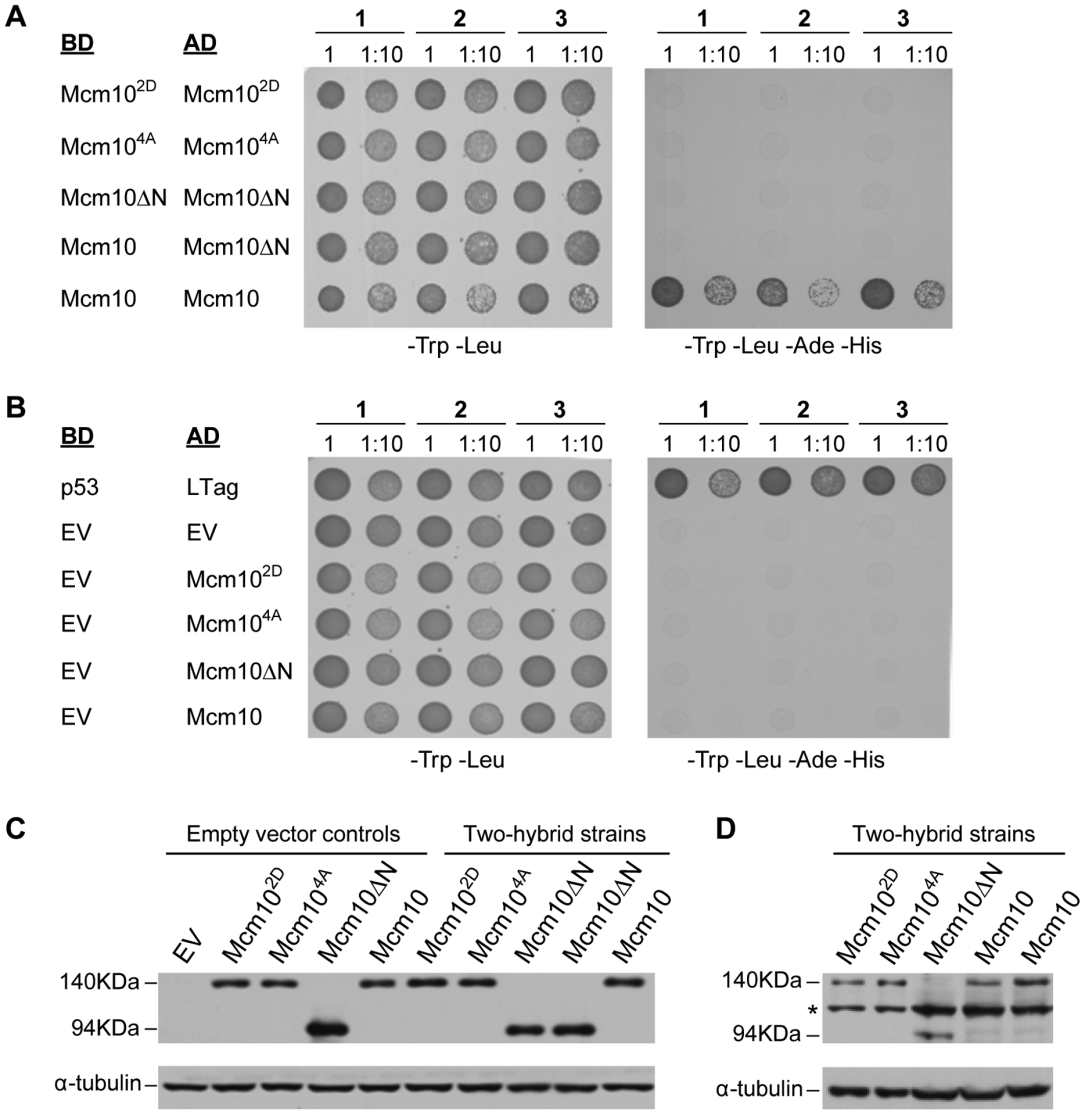
Coiled-coil mutations disrupt Mcm10 self-association. (A, B) Three individual strains (1, 2, 3) harboring yeast two-hybrid plasmids that express the indicated proteins either as a fusion with the Gal4-binding domain (BD) or -activation domain (AD) were spotted onto drop-out plates lacking tryptophan and leucine (-Trp -Leu) or quadruple drop-out plates lacking tryptophan, leucine, adenine and histidine (-Trp -Leu -Ade -His). Cells were spotted at a number of 2×10^7^ (1) or a 10-fold dilution (1∶10) for full-length *Xenopus* Mcm10, the L104D/L108D mutant (Mcm10^2D^), the L104A/L108A/M115A/L118A mutant (Mcm10^4A^), and the NTD deletion mutant spanning residues 230–860 (Mcm10ΔN). p53 and large T-antigen (LTag) served as a positive control, EV indicates empty vector controls. (C) Western blot showing wild-type and mutant xMcm10 protein expression in representative strains. Gal4-AD fusions were detected by a HA-specific antibody. Strains carrying empty vector controls, or Gal4-AD fusion genes and Gal4-BD empty vectors are shown on the left (Empty vector controls). Strains expressing pair-wise combinations of the Gal4-AD and Gal4-BD fusion genes as indicated in panels A and B are shown on the right (Two-hybrid strains). Full-length xMcm10 and the 2D and 4A mutants ran at an approximate size of 140 kDa, whereas the truncated form of xMcm10 ran at an approximate size of 94 kDa. Tubulin served as a loading control. (D) Gal4-BD fusions were detected by a Myc-specific antibody. Extracts from the identical two-hybrid strains shown in (C) were loaded in the same order. The asterisk denotes a non-specific band.

To confirm that the 2D and 4A mutations impeded Mcm10 dimerization *in vivo*, we conducted a yeast two-hybrid analysis (Figure 6). Full-length xMcm10 as well as the 2D, 4A and Mcm10ΔN mutants were each fused to either a Gal4-binding or -activation domain. The interaction between T-antigen (T-ag) and p53 served as a positive control, whereas combinations of the respective activation domain fusions combined with an empty vector served as negative controls. Plasmid retention was evaluated by spotting cells onto double selection plates lacking leucine and tryptophan. We tested three independent strains for each of the two-hybrid pairs, as indicated in Figures 6A and B. The ability to interact was scored on quadruple selection plates. Full-length xMcm10 displayed strong self-interaction, almost at the level as the binding between T-ag and p53 (compare Figures 6A and B, right panels). In contrast, the interaction was eliminated by the 2D and 4A point mutations and the N-terminal deletion construct (Figure 6A). As expected, empty vector controls did not show any viable colonies (Figure 6B). Importantly, the lack of self-association between the respective mutants or the N-terminally truncated protein and full-length xMcm10 was not due to differences in protein expression, as analyzed by Western blot (Figure 6C,D). Since we also observed a significant difference in the sedimentation velocity profile of purified full-length wild-type protein and the 2D mutant (Figure S2B), we conclude that the CC is the primary oligomerization motif in xMcm10.

## Discussion

This work identifies an evolutionarily conserved CC motif in the N-terminus of xMcm10 and provides evidence that it is required for self-association. Our data also strongly suggest that Mcm10 exists in a dynamic equilibrium between multiple oligomeric states, which helps to explain the disagreement in the literature regarding the number of subunits. We observed a broad distribution of states of the full-length protein using two quantitative approaches, and consistently found the presence of both dimeric and trimeric species. It is striking that in addition to estimates of dimer and trimer formation of full-length xMcm10 by SEC-MALS, we detected a mixture of dimeric and trimeric forms of the isolated CC region, with dimers predominating in solution and a trimer in crystals.

The propensity of the Mcm10 CC to form multiple states can be explained by the particular CC sequence, since the rules governing the number of CC subunits as a function of the amino acids at the *a* and *d* positions within the heptad repeat is well understood [56], [57], [59], [61]. Inclusion of methionine at the *a* position in the human, mouse, and frog Mcm10 CC (Met115 in our structure) likely destabilizes the dimer and would even favor parallel tetramers and pentamers [61]. This raises the possibility that the Mcm10 CC could accommodate higher order oligomers, formed either as a simple association between the helices or as more complex patterns such as a trimer of dimers [62], [63], [64]. Regardless of the oligomeric state, the residues lining the supercoil interface would remain the same. Indeed, our data indicate that the *a* and *d* residues identified in the trimer crystal structure are important for dimerization of the CC and the NTD *in vitro* and the self-association of the full-length protein *in vivo*.

The existence of dimers and trimers implies that the Mcm10 CC is metastable and therefore its oligomeric state is sensitive to environmental factors. In support of this, the Mcm10 CC trimer is stabilized at lower pH. Interestingly, pH dependent CC switches are important biological mechanisms by which proteins change conformation to drive various processes [56]. For example, viral glycoproteins adopt trimeric CCs in response to pH as a mechanism to fuse viral and cellular membranes [63], [65], [66]. In fact, the crystal structure of the human T cell leukemia virus type 1 transmembrane ectodomain, determined as an MBP fusion, formed a parallel trimeric CC required for proper function [67], [68], further validating the importance of our trimeric MBP-Mcm10-CC structure. In addition, CC folding and remodeling in response to other environmental factors, including temperature and effector molecules is a general phenomenon [69], [70], [71], [72], [73], [74], [75]. On the basis of these examples and consistent with our data, we speculate that the Mcm10 CC exists mainly as an intrinsically disordered monomer or as a CC dimer, and has the propensity to attain other multimeric configurations in response to its environment.

It is intriguing to speculate that Mcm10 may adopt different oligomeric states to perform multiple roles during DNA replication. For example, higher-order oligomers may be used for sequestering Mcm10 at the replication fork. Upon pre-RC activation and origin melting, Mcm10 may reform as a dimer or trimer as DNA is denatured and replication factors recruited to the emerging fork. scMcm10 was reported to exhibit differential packing on ssDNA versus dsDNA [21], suggesting that a change in the Mcm10 conformation or its oligomeric state could facilitate strand separation. In this context, it is noteworthy that Mcm10 binds ssDNA with a 3–5-fold preference over dsDNA [19], [20], [21]. Oligomerization on ssDNA might thus assist in the initial unwinding step and aid what has been termed “helicase activation” [3] but may very well just be the coordinated stabilization of short stretches of unwound DNA after the separation of Mcm2–7 dimers [11], [76].

Mcm10’s modular architecture and lack of enzymatic activity suggest that it serves as a scaffold to orchestrate protein and DNA interactions within the replisome. Self-association would provide multiple points of contact between replication factors and DNA [37]. Indeed, Mcm10 is involved in multiple interactions, including but not limited to the replication and checkpoint clamps, PCNA and 9-1-1 [unpublished results and ref. 35], and pol α [16], [17], [20], [33], [34]. Protein-protein interactions could be mediated by the CC directly, similar to the interaction between Cdt1 and geminin [77], [78], [79]. Alternatively, dimerization could facilitate molecular interactions and recruiting proteins to the origin simply by increasing the number of possible binding sites on Mcm10 [37]. For example, the ID and CTD each bind DNA and pol α and could therefore be involved in a molecular hand-off, whereby Mcm10 is anchored to DNA via the ID while binding pol α at the CTD, and *vice versa* [34]. Additional Mcm10 subunits would enhance these interactions by increasing the number of ID and CTD present. Similarly, a parallel Mcm10 dimer could couple events on the leading and lagging strands or physically tether the helicase and pol α [16], [18], [27] while retaining the polarity necessary for fork progression. This would also explain why loss of the first 100 residues of scMcm10 confers such a strong sensitivity to hydroxyurea in the absence of the 9-1-1 clamp (Alver and Bielinsky, unpublished results).

Taken together, the dimerization or trimerization of xMcm10 in the absence of DNA reported here is consistent with previous work on spMcm10 [42], and the observation that three subunits of scMcm10 are bound to short ssDNA oligonucleotides, although these latter complexes were not shown to have the three-fold symmetry revealed in our crystal structure [21]. We did not find any evidence for Mcm10 hexamers, which were previously observed by EM of the human protein [41]. As discussed above we do not rule out a trimer of dimers, although this would not be consistent with the six-fold symmetrical EM structure reported.

## Supporting Information

Figure S1 **Dimerization of the putative Mcm10 coiled-coil region.** (A,B) Sedimentation velocity profiles of free MBP (A) and MBP-CC^95–132^ (B) at pH 7.4. Molecular masses derived from the data (Table S1) are 43 kDa (MBP) and 52 and 75 kDa (MBP-CC), corresponding to 1.2 and 1.7 MBP-CC subunits, respectively. (C) SDS-PAGE of MBP-CC^95–132^ in the presence of varying amounts of reducing agents. Both bands were confirmed by mass spectrometry to be xMcm10 residues 95–132. The loading buffer in each sample contained 62.5 mM Tris-HCl (pH 6.8), 10% glycerol, 2% SDS (w/v), and bromophenol blue in addition to the reducing agents shown at the top of each lane. The peak marked with an asterisk (*) represents a molecular mass exactly twice that of the calculated mass, and persisted at DTT concentrations as high as 200mM (not shown).
(TIF)

Figure S2 **Effect of coiled-coil point mutants on Mcm10 self-association.** (A) Native gel electrophoresis (4–16% Bis-Tris) of the NTD as wild-type (WT), 2A (L104A/L108A), 2D (L104D/L108D), or 4A (L104A/L108A/M115A/L118A). Size markers in kDa are shown to the left. (B) Sedimentation velocity analytical ultracentrifugation of full-length Mcm10 (black, wild-type; blue, 2D mutant). Data were collected at 4°C and 42,000 rpm in PBS buffer, 150 mM NaCl, and 0.3 mM TCEP at protein concentrations of 1.6 mg/ml (WT) and 1.0 mg/ml (2D). The estimated masses of these peaks are shown in Table S1. Although the precise masses cannot be accurately determined due to the complex nature of the sedimentation profile, the reduction of the 4S peak (marked with an asterisk) in the monomeric 2D mutant represents a significant difference from the WT.
(TIF)

Table S1 **Sedimentation velocity data for Mcm10 constructs.**
(PDF)

Table S2 **Crystallographic data collection and refinement statistics for MBP-CC^95–124^.**
(PDF)
