# Supplementary figures and images for: Mcm10 Self-Association Is Mediated by an N-Terminal Coiled-Coil Domain
Source: PLoS One. 2013 Jul 23;8(7):e70518. doi: 10.1371/journal.pone.0070518 (PMC3720919; doi:10.1371/journal.pone.0070518)

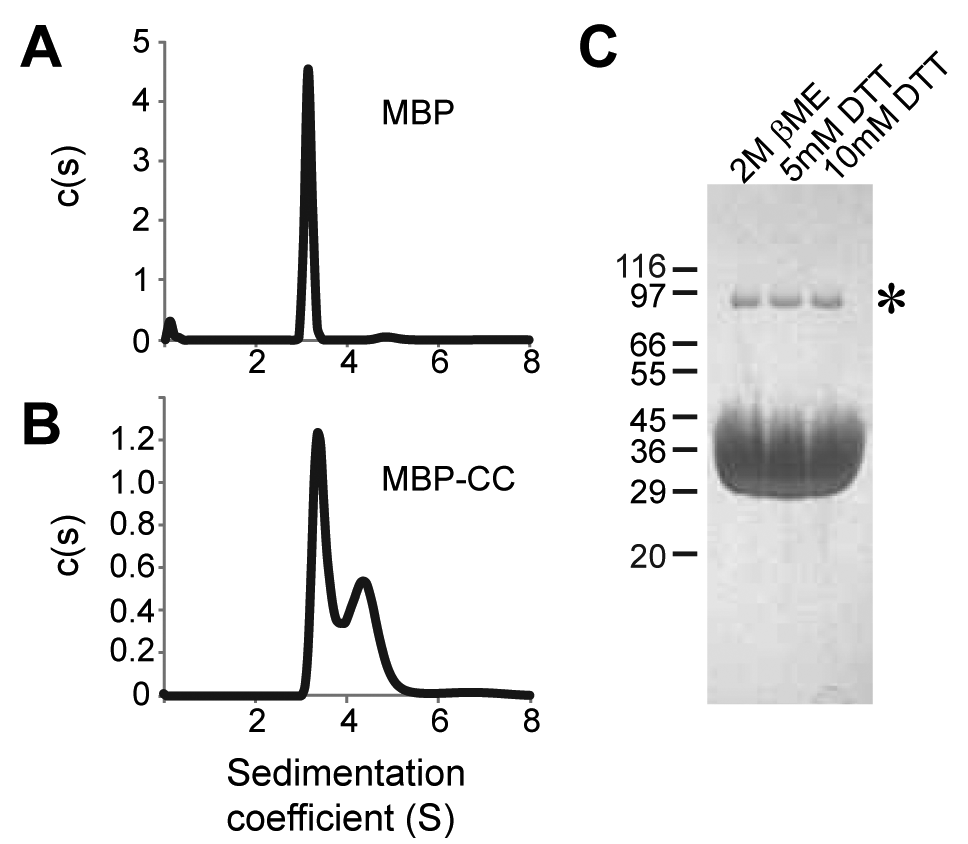

Supplement: Figure S1 — Dimerization of the putative Mcm10 coiled-coil region. (A,B) Sedimentation velocity profiles of free MBP (A) and MBP-CC95–132 (B) at pH 7.4. Molecular masses derived from the data (Table S1) are 43 kDa (MBP) and 52 and 75 kDa (MBP-CC), corresponding to 1.2 and 1.7 MBP-CC subunits, respectively. (C) SDS-PAGE of MBP-CC95–132 in the presence of varying amounts of reducing agents. Both bands were confirmed by mass spectrometry to be xMcm10 residues 95–132. The loading buffer in each sample contained 62.5 mM Tris-HCl (pH 6.8), 10% glycerol, 2% SDS (w/v), and bromophenol blue in addition to the reducing agents shown at the top of each lane. The peak marked with an asterisk (*) represents a molecular mass exactly twice that of the calculated mass, and persisted at DTT concentrations as high as 200mM (not shown). (TIF) [file pone.0070518.s001.tif]

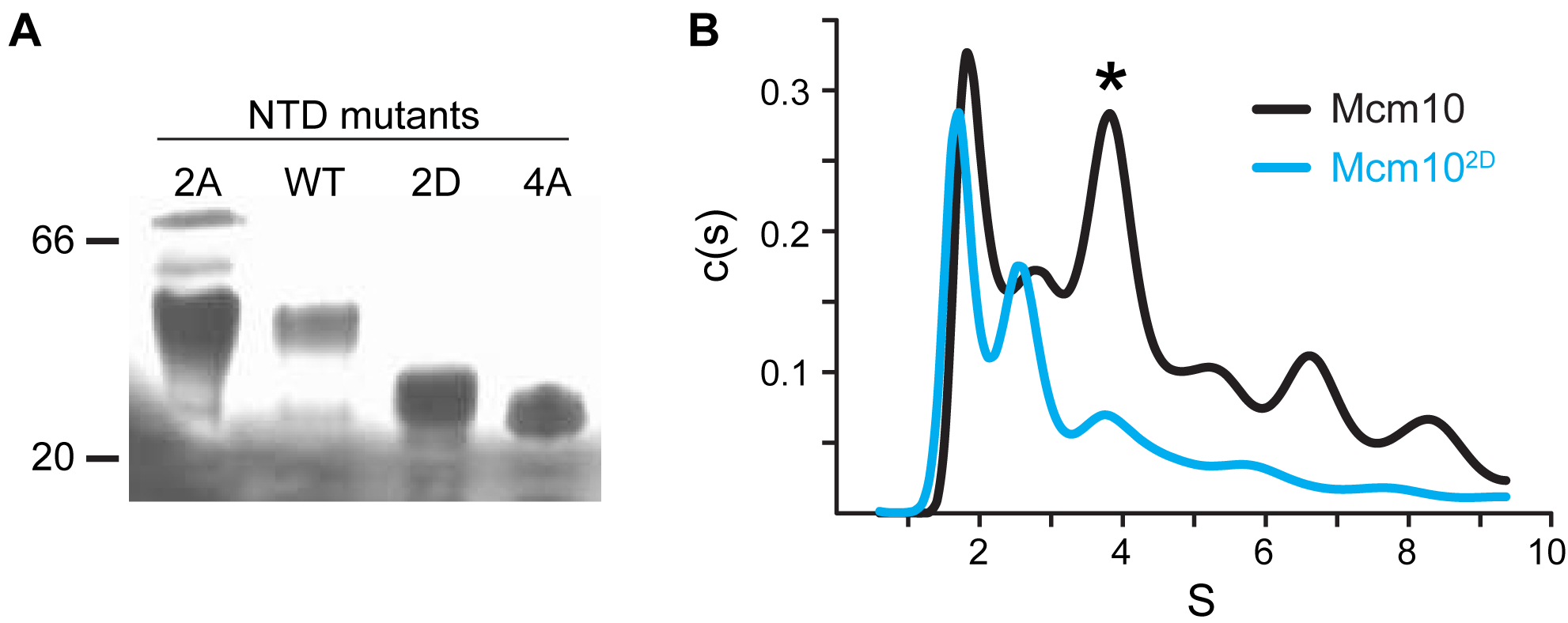

Supplement: Figure S2 — Effect of coiled-coil point mutants on Mcm10 self-association. (A) Native gel electrophoresis (4–16% Bis-Tris) of the NTD as wild-type (WT), 2A (L104A/L108A), 2D (L104D/L108D), or 4A (L104A/L108A/M115A/L118A). Size markers in kDa are shown to the left. (B) Sedimentation velocity analytical ultracentrifugation of full-length Mcm10 (black, wild-type; blue, 2D mutant). Data were collected at 4°C and 42,000 rpm in PBS buffer, 150 mM NaCl, and 0.3 mM TCEP at protein concentrations of 1.6 mg/ml (WT) and 1.0 mg/ml (2D). The estimated masses of these peaks are shown in Table S1. Although the precise masses cannot be accurately determined due to the complex nature of the sedimentation profile, the reduction of the 4S peak (marked with an asterisk) in the monomeric 2D mutant represents a significant difference from the WT. (TIF) [file pone.0070518.s002.tif]
