# Supplementary material for: Mcm10 Self-Association Is Mediated by an N-Terminal Coiled-Coil Domain
Source: PLoS One. 2013 Jul 23;8(7):e70518. doi: 10.1371/journal.pone.0070518 (PMC3720919; doi:10.1371/journal.pone.0070518)
Supplement: Table S1 — Sedimentation velocity data for Mcm10 constructs. (PDF) [file pone.0070518.s003.pdf]

Table S1. Sedimentation velocity data for Mcm10 constructs

	pH	Conc (mg/mL)	S	$S_{20,w}$ [1]	MW_{calc} [2]	MW_{exp} [3]	# subunits [4]	Peak area
**Mcm10**								
WT	7.4	1.7	1.98	3.17	95.4	52.8	0.5	19.7
	7.4	1.7	2.87	4.58	95.4	91.6	1.0	13.5
	7.4	1.7	3.96	6.33	95.4	149	1.6	27.9
2D	7.4	1.0	1.76	2.80	95.4	42.2	0.4	30.2
	7.4	1.0	2.66	4.24	95.4	78.8	0.8	28.4
	7.4	1.0	4.19	6.67	95.4	156	1.6	19.7
**Mcm10ΔN**								
WT	7.4	1.0	1.53	2.99	70.4	68.8	1.0	74.1
	7.4	1.0	2.75	5.38	70.4	166.0	2.4	19.2
	7.4	1.6	1.53	2.99	70.4	71.6	1.0	71.2
	7.4	1.6	2.55	4.99	70.4	152.0	2.2	17.3
	7.4	3.5	1.36	2.66	70.4	71.2	1.0	79.3
	7.4	3.5	2.32	4.55	70.4	160.0	2.3	18.0
**NTD**								
WT	7.4	0.4	1.62	1.59	16.0	27.5	1.7	76.4
2D	7.4	0.4	1.30	1.26	16.0	13.2	0.8	78.4
4A	7.4	0.4	1.31	1.27	15.8	17.0	1.1	71.4
**Coiled-coil**								
$MBP\text{-}CC^{95\text{-}132}$	7.4	0.6	3.48	3.61	45.1	52.4	1.2	55.2
	7.4	0.6	4.41	4.58	45.1	74.8	1.7	42.8
	4.7	0.6	3.48	3.61	45.1	46.0	1.0	7.5
	4.7	0.6	5.17	5.36	45.1	84.1	1.9	77.3
	4.7	0.6	6.84	7.10	45.1	129.0	2.9	14.0
$MBP\text{-}CC^{95\text{-}124}$ WT	7.4	0.7	3.22	3.50	44.3	52.2	1.2	27.3
	7.4	0.7	4.79	5.21	44.3	94.7	2.1	65.8
$MBP\text{-}CC^{95\text{-}124}$ 2D	7.4	0.7	3.15	3.42	44.1	49.1	1.1	96.9
$MBP\text{-}CC^{95\text{-}124}$ 4A	7.4	0.6	3.15	3.41	43.9	45.4	1.0	98.5
MBP	7.4	0.6	3.14	3.46	40.4	43.0	1.1	92.0
	4.7	0.6	3.26	3.55	40.4	42.1	1.0	99.5

[1] Sedimentation coefficient at standard conditions (water at 20°C).
[2] MW_{calc} is the molecular weight in kDa calculated from the amino acid composition
[3] MW_{exp} is the molecular weight in kDa derived from the ultracentrifugation data
[4] # subunits = MW_{exp}/ MW_{calc}
