# Supplementary material for: Mcm10 Self-Association Is Mediated by an N-Terminal Coiled-Coil Domain
Source: PLoS One. 2013 Jul 23;8(7):e70518. doi: 10.1371/journal.pone.0070518 (PMC3720919; doi:10.1371/journal.pone.0070518)
Supplement: Table S2 — Crystallographic data collection and refinement statistics for MBP-CC95–124. (PDF) [file pone.0070518.s004.pdf]

**Table S2.** Crystallographic data collection and refinement statistics for MBP-CC$^{95\text{-}124}$

| | |
|---|---|
| **Data collection** | |
| Wavelength (Å) | 0.97872 |
| Space group | C2 |
| Cell dimensions | |
| *a, b, c* (Å) | 187.4, 116.6, 73.4 |
| α, β, γ (°) | 90, 103.5, 90 |
| Resolution (Å) | 30.0-2.4 (2.5-2.4) |
| $R_{sym}$ | 0.069 (0.401) |
| $I / \sigma_I$ | 23.2 (3.6) |
| Completeness (%) | 98.6 (94.4) |
| Redundancy | 7.1 (6.4) |
| | |
| **Refinement** | |
| Resolution (Å) | 30-2.4 |
| Unique reflections | 59,488 |
| $R_{work}$ / $R_{free}$ | 0.164 / 0.205 |
| No. of atoms | |
| Protein/maltose | 8,934 |
| Solvent | 446 |
| Ave. B-factors | |
| Protein/maltose | 46.4 |
| Solvent | 42.8 |
| R.m.s. deviations | |
| Bond lengths (Å) | 0.008 |
| Bond angles (°) | 1.105 |
| PDB ID | 4JBZ |

Values in parentheses refer to the highest resolution shell.
